# Assessing the Influence of Lamina Terminalis Opening in Aneurysm Surgery: Clinical Outcomes and Ventriculoperitoneal Shunt Insertion Rates

**DOI:** 10.7759/cureus.99425

**Published:** 2025-12-16

**Authors:** Ali Ayyad, Muhammad Mohsin Khan, Ali Msheik, Noman Shah, Muath Hussein, Ahmed Eid, Nisar Sheikh

**Affiliations:** 1 Neurosurgery, Weill Cornell Medical College, Doha, QAT; 2 Neurosurgery, Jordan University of Science and Technology, Doha, QAT; 3 Clinical Research, University of Dresden, Dresden, DEU; 4 Neurological Surgery, Hamad Medical Corporation, Doha, QAT; 5 Neurosurgery, University of Arizona College of Medicine, Arizona, USA; 6 Surgical Intensive Care Unit (SICU), Hamad Medical Corporation, Doha, QAT

**Keywords:** aneurysmal subarachnoid hemorrhage, aneurysm surgery, hydrocephalus, lamina terminalis opening, microsurgical clipping, ventriculoperitoneal shunt

## Abstract

Background

Opening the lamina terminalis during microsurgical clipping of anterior communicating artery (AComA) aneurysms has been proposed to reduce postoperative hydrocephalus and shunt dependency.

Methods

We retrospectively reviewed 113 patients who underwent microsurgical clipping for aneurysmal subarachnoid hemorrhage (aSAH). Patient demographics, operative notes, and outcomes were collected. The primary endpoint was ventriculoperitoneal shunt (VPS) placement, and the secondary endpoint was postoperative hydrocephalus. Outcomes were compared between patients with and without lamina terminalis opening using descriptive statistics and Fisher’s exact test.

Results

The lamina terminalis was opened in 84 patients (74.3%). VPS placement was required in four patients overall, all from the fenestration group (4/84, 4.8% vs 0/29, 0%; p = 0.57). Hydrocephalus occurred in 30 patients with fenestration (35.7%) and 10 without (34.5%; p = 1.00). No significant differences were observed between groups for either endpoint.

Conclusion

In this single-center cohort, no measurable difference was observed. These findings suggest that fenestration may be useful intraoperatively for cerebrospinal fluid release and exposure, but it should not be relied upon as a preventive measure against long-term shunt dependence.

## Introduction

Aneurysmal subarachnoid hemorrhage (aSAH) is a devastating emergency with high mortality and morbidity despite modern microsurgical and endovascular advances [1]. Despite advances in microsurgical and endovascular techniques, aSAH continues to carry high morbidity and mortality [2]. Hydrocephalus develops in up to one-third of patients after aSAH and has been associated with poor neurological outcome and long-term hospitalization [3]. Treatment of hydrocephalus includes the insertion of a ventriculoperitoneal shunt (VPS), which itself carries risks such as infection, obstruction, and shunt dependency [4].

Lamina terminalis opening has been advocated as an interoperative technique to facilitate CSF drainage, reduce brain retraction, and, importantly, the development of postoperative hydrocephalus [5-7]. Previous studies have reported conflicting results regarding whether lamina terminalis opening reduces postoperative hydrocephalus [8-13].

## Materials and methods

This retrospective observational study included consecutively admitted patients who underwent microsurgical clipping for anterior communicating artery (AComA) aneurysms at our tertiary neurosurgical center. 

Inclusion and exclusion criteria

Patients were eligible if they presented with AComA SAH confirmed radiographically by computed tomography angiography (CTA) or digital subtraction angiography (DSA) and were subsequently treated by microsurgical clipping of the AComA aneurysm. The inclusion period was from January 2016 to October 2025. 

Data collection

Demographic variables collected included age, sex, and nationality. Clinical severity at presentation was extracted from admission records, including the World Federation of Neurosurgical Societies (WFNS) grade, Glasgow Coma Scale (GCS) score, and Fisher hemorrhage grade on initial CT imaging. Additional perioperative variables included postoperative GCS at 24 hours, length of neurosurgical intensive care observation, and the time interval between aneurysm clipping and any subsequent VPS insertion.

Operative reports were reviewed to determine whether lamina terminalis fenestration was performed during aneurysm clipping. The decision to fenestrate the lamina terminalis was based solely on intraoperative surgical judgment and was not standardized by protocol.

Hydrocephalus was assessed both radiographically and clinically. Radiologic hydrocephalus was defined as new or progressive ventricular enlargement on postoperative CT scans. Symptomatic hydrocephalus was defined by compatible clinical features such as impaired consciousness, external ventricular drainage (EVD) weaning failure, gait disturbance, or cognitive decline. For patients requiring permanent CSF diversion, the timing of radiologic hydrocephalus onset and the timing of symptomatic progression were recorded. The need for additional neurosurgical interventions, including VPS placement, was documented throughout hospitalization and early follow-up.

Follow-up and outcomes

All patients were followed clinically until discharge from the hospital. Outpatient follow-up notes and radiologic studies were subsequently reviewed to identify any cases of delayed or chronic hydrocephalus necessitating VPS insertion. The primary outcome measure was the requirement for permanent CSF diversion (VPS placement). The secondary outcome was the development of postoperative hydrocephalus, whether transient or persistent.

Statistical analysis

Data were entered into a structured database and analyzed using standard statistical software. Descriptive statistics were used to summarize baseline demographic and clinical characteristics. Continuous variables were expressed as means ± standard deviation (SD) or medians with interquartile ranges (IQR), depending on data distribution. Categorical variables were compared using Fisher’s exact test, and continuous variables were analyzed using appropriate parametric (Student’s t-test) or nonparametric (Mann-Whitney U test) tests. A p-value < 0.05 was considered statistically significant.

Ethical considerations

Ethical approval for this retrospective study was obtained from the Institutional Review Board of Hamad Medical Corporation (HMC), Doha, Qatar (Approval Number: MRC-01-25-1136). The study was conducted in accordance with the ethical principles outlined in the Declaration of Helsinki (1964) and its subsequent amendments. Patient confidentiality was strictly maintained, and no identifying information was included in the study database or subsequent analyses.

## Results

The mean age of the cohort was 45 years (range: 26-72), and 60% were males. Lamina terminalis fenestration was performed in 84 patients (74.3%). The baseline clinical severity was moderate: the median WFNS grade was 3 (IQR: 2-4), the median GCS score on admission was 12 (IQR: 9-14), and Fisher grades were distributed as follows: Fisher 2 in 18.6%, Fisher 3 in 54.3%, and Fisher 4 in 27.1% of patients. The median cisternal hematoma volume measured on admission CT was 18 mL (IQR: 12-28).

Postoperative observation and early neurologic course

All patients underwent postoperative monitoring in the neurosurgical ICU for a minimum of 10 days, with a mean observation period of 12.4 ± 3.1 days. Neurological status modestly improved during the early postoperative period, with the median GCS rising to 13 (IQR: 11-15) at 24 hours after aneurysm clipping. EVD was required in 32 patients (28.3%), with a median duration of six days (IQR: 4-8). Patients who later developed hydrocephalus tended to have longer EVD duration (median 7 vs. 5 days), although this did not reach statistical significance (p = 0.08).

Hydrocephalus incidence

Hydrocephalus was diagnosed in 30 of 84 patients who underwent lamina terminalis opening (35.7%) and in 10 of 29 patients without fenestration (34.5%). There was no significant difference between groups (p = 1.00; odds ratio: 0.95; 95% CI: 0.40-2.37). Radiologic hydrocephalus typically developed within five days of subarachnoid hemorrhage onset (IQR: 3-7), while symptomatic hydrocephalus manifested at a median of seven days (IQR: 6-9). Patients with Fisher grade 4 hemorrhage and larger initial cisternal hematoma volumes exhibited a higher likelihood of hydrocephalus development.

Shunt dependency

VPS placement was required in four patients (3.5%), all of whom were in the fenestration group (p = 0.57). These patients demonstrated markers of greater hemorrhage severity, including larger initial hematoma volumes (median: 26 mL; IQR: 20-33), lower admission GCS scores (median: 9; IQR: 7-11), and Fisher grade 4 hemorrhage in 75% of cases. The median interval between aneurysm clipping and VPS placement was 14 days (range: 10-21). No cases of delayed or late-onset hydrocephalus occurred after discharge (Table 1).

**Table 1:** Patient characteristics and outcomes by lamina terminalis opening * p-values calculated using independent t-test for continuous variables and Fisher’s exact test for categorical variables. ^† ^Fisher’s exact test

Variable	Lamina opened (n = 84)	Lamina not opened (n = 29)	p-value*
Mean age (years)	45.7	45.5	0.91
Male sex	51 (60.7%)	17 (58.6%)	0.84†
Hydrocephalus	30 (35.7%)	10 (34.5%)	1.00†
Ventriculoperitoneal shunt	4 (4.8%)	0 (0.0%)	0.57†
External ventricular drain	24 (28.6%)	7 (24.1%)	0.80†

Established predictors such as intraventricular blood burden and the need for external ventricular drainage are likely more important determinants of outcome.

## Discussion

Shunt insertion occurred in only four patients overall, all from the fenestration group (4.8% vs 0%), but this difference was not statistically significant. These results indicate that fenestration did not provide a protective effect against either outcome in this cohort.

Our findings are consistent with several prior reports that have also failed to demonstrate a reduction in shunt dependence after lamina terminalis opening [7,9,12,14]. While some investigators have described modest benefits, those associations were variable between centers and often attenuated when adjusted for clinical severity and intraventricular blood burden [12,14]. The accumulated evidence therefore suggests that fenestration is not a major determinant of long-term CSF dynamics after aSAH.

Biological considerations help explain these neutral findings. Hydrocephalus after aSAH arises from impaired CSF absorption and fibrosis, processes unlikely to be altered by fenestration. These diffuse processes are unlikely to be modified by a single intraoperative fenestration [1,11].

Several limitations of this study must be acknowledged. Confounding by indication is likely. We were unable to adjust for important predictors such as Fisher grade, intraventricular blood burden, and EVD duration. This residual confounding limits causal interpretation. Second, although inpatient and follow-up records were reviewed, the study may not have captured all late cases of shunt-dependent hydrocephalus.

Despite the absence of long-term protective benefit, fenestration may still hold intraoperative value. It can facilitate CSF release, improve brain relaxation, and provide safer exposure during aneurysm clipping. However, the procedure should not be considered a strategy for preventing chronic hydrocephalus or shunt dependence. Decisions about CSF diversion must instead be guided by the postoperative course and radiographic findings. Future research should focus on larger multicentre registries or pragmatic randomized trials with standardized definitions, perioperative protocols, and detailed recording of clot burden and drainage practices. 

## Conclusions

This maneuver remains useful for exposure and brain relaxation but should not be selected to prevent long-term shunt dependence. Future studies should use richer clinical covariates, multicenter cohorts, and pragmatic randomization when feasible to define indications with confidence.

Although our data do not support a protective effect against hydrocephalus, lamina terminalis opening continues to be a technically simple step that can assist surgeons intraoperatively when the brain is tense or the operative corridor is narrow. In centers where fenestration is routinely practiced, its use should be based on the surgeon's judgment rather than an expectation of reducing shunt rates. These findings may help guide realistic intraoperative decision-making and prevent unnecessary reliance on this technique for cerebrospinal fluid diversion prevention.

## References

[1] Connolly ES Jr, Rabinstein AA, Carhuapoma JR (2012). Guidelines for the management of aneurysmal subarachnoid hemorrhage: a guideline for healthcare professionals from the American Heart Association/american Stroke Association. Stroke.

[2] Macdonald RL, Schweizer TA (2017). Spontaneous subarachnoid haemorrhage. Lancet.

[3] Chen S, Luo J, Reis C, Manaenko A, Zhang J (2017). Hydrocephalus after subarachnoid hemorrhage: pathophysiology, diagnosis, and treatment. Biomed Res Int.

[4] Desai VR, Sadrameli SS, Jenson AV, Asante SK, Daniels B, Trask TW, Britz G (2020). Ventriculoperitoneal shunt complications in an adult population: a comparison of various shunt designs to prevent overdrainage. Surg Neurol Int.

[5] Dostovic A, Moranjkic M, Galijasevic K, Mujezinovic A, Salihovic D, Kunic S (2025). Complications and outcome in patients with hydrocephalus who have had a ventriculoperitoneal shunt implanted. Med Arch.

[6] Ibrahim TF, Hafez A, Andrade-Barazarte H (2015). De novo giant A2 aneurysm following anterior communicating artery occlusion. Surg Neurol Int.

[7] Komotar RJ, Olivi A, Rigamonti D, Tamargo RJ (2002). Microsurgical fenestration of the lamina terminalis reduces the incidence of shunt-dependent hydrocephalus after aneurysmal subarachnoid hemorrhage. Neurosurgery.

[8] Sindou M (1994). Favourable influence of opening the lamina terminalis and Lilliequist's membrane on the outcome of ruptured intracranial aneurysms. A study of 197 consecutive cases. Acta Neurochir (Wien).

[9] Veldeman M, Höllig A, Clusmann H, Stevanovic A, Rossaint R, Coburn M (2016). Delayed cerebral ischaemia prevention and treatment after aneurysmal subarachnoid haemorrhage: a systematic review. Br J Anaesth.

[10] Vergouwen MD (2011). Magnesium sulfate for aneurysmal subarachnoid hemorrhage: the end of the road or more trials?. Crit Care.

[11] de Sousa AA, Dantas FL, de Cardoso GT, Costa BS (1999). Distal anterior cerebral artery aneurysms. Surg Neurol.

[12] Konczalla J, Platz J, Schuss P, Vatter H, Seifert V, Güresir E (2014). Non-aneurysmal non-traumatic subarachnoid hemorrhage: patient characteristics, clinical outcome and prognostic factors based on a single-center experience in 125 patients. BMC Neurol.

[13] Giussani C, Di Cristofori A (2021). Lamina terminalis fenestration: an important neurosurgical corridor. Handb Clin Neurol.

[14] Tomasello F, d'Avella D, de Divitiis O (1999). Does lamina terminalis fenestration reduce the incidence of chronic hydrocephalus after subarachnoid hemorrhage?. Neurosurgery.

